# P-154. Malaria In Tunisia : Eradicated Or Still Endemic ?

**DOI:** 10.1093/ofid/ofae631.359

**Published:** 2025-01-29

**Authors:** Fatma Hammami, Khaoula Rekik, Fatma Smaoui, Amal Chakroun, Chakib Marrakchi, Makram Koubaa, Mounir Ben Jemaa

**Affiliations:** Infectious Diseases Department, Hedi Chaker University Hospital, University of Sfax, Tunisia, Sfax, Sfax, Tunisia; Infectious Diseases Department, Hedi Chaker University Hospital, University of Sfax, Tunisia, Sfax, Sfax, Tunisia; Infectious Diseases Department, Hedi Chaker University Hospital, University of Sfax, Tunisia, Sfax, Sfax, Tunisia; Infectious Diseases Department, Hedi Chaker University Hospital, University of Sfax, Tunisia, Sfax, Sfax, Tunisia; Infectious Diseases Department, Hedi Chaker University Hospital, University of Sfax, Tunisia, Sfax, Sfax, Tunisia; Infectious Diseases Department, Hedi Chaker University Hospital, University of Sfax, Tunisia, Sfax, Sfax, Tunisia; Infectious Diseases Department, Hedi Chaker University Hospital, University of Sfax, Tunisia, Sfax, Sfax, Tunisia

## Abstract

**Background:**

Although malaria has been eradicated in Tunisia since 1979, our country remains exposed to the potential risk of resurgence. We aimed to study clinical, laboratory and therapeutic features of malaria.

**Methods:**

We conducted a retrospective study including all patients hospitalized in the infectious disease department for malaria between 2000 and 2022.

**Results:**

We encountered 92 cases, among whom 85 were males (92.4%). The mean age was 30±11 years. Fifteen patients received malaria chemoprophylaxis (16.3%). Returned travellers developed symoptoms after 10[5-25] days of returning home. Countries involved in importation were Ivory Coast (31.5%), Mauritania (8.9%) and Senegal (6.5%). The revealing symptoms included fever (90.2%), chills (87%), cephalalgia (79.3%) and diarrhea (26.1%). Arthromyalgia and vomiting were noted 46 cases (50%), each. Thrombopenia (65.2%), elevated C-reactive protein levels (43.5%), lymphopenia (39.1%), anemia (31.5%) and hepatic cytolysis (27.1%) were reported. There were 23 cases of severe malaria (25%). *Palsmodium falciparum* was identified in 68 cases (74%) and *Plasmodium vivax* in 8 cases (8.9%). Gametocytes were detected in 8.9% of the cases. Patients were treated with artemether/lumefantrine (71.7%) or quinine (19.6%). The disease evolution was favorable in 90 cases (97.8%). Apyrexia was noted after 2±1 days of treatment. Two patients were dead (2.2%).

**Conclusion:**

Travellers to malaria endemic countries should be aware of the risk of malaria in order to follow preventive measures and to promptly consult in case of symptoms. An early diagnosis followed by the adequate treatment might prevent severe forms and death.

**Disclosures:**

**All Authors**: No reported disclosures

